# Ethylene responsive factors in the orchestration of stress responses in monocotyledonous plants

**DOI:** 10.3389/fpls.2015.00640

**Published:** 2015-08-28

**Authors:** Sanjukta Dey, A. Corina Vlot

**Affiliations:** Helmholtz Zentrum Muenchen, Department of Environmental Sciences, Institute of Biochemical Plant Pathology, Neuherberg, Germany

**Keywords:** AP2/ERFs, ethylene-responsive factors, biotic stress, abiotic stress, monocot

## Abstract

The APETALA2/Ethylene-Responsive Factor (AP2/ERF) superfamily of transcription factors (TFs) regulates physiological, developmental and stress responses. Most of the AP2/ERF TFs belong to the ERF family in both dicotyledonous and monocotyledonous plants. ERFs are implicated in the responses to both biotic and abiotic stress and occasionally impart multiple stress tolerance. Studies have revealed that *ERF* gene function is conserved in dicots and monocots. Moreover, successful stress tolerance phenotypes are observed on expression in heterologous systems, making ERFs promising candidates for engineering stress tolerance in plants. In this review, we summarize the role of ERFs in general stress tolerance, including responses to biotic and abiotic stress factors, and endeavor to understand the cascade of ERF regulation resulting in successful signal-to-response translation in monocotyledonous plants.

## Introduction

The APETALA2/Ethylene-Responsive Factor (AP2/ERF) superfamily of transcription factors (TFs) regulates diverse plant responses, from light acclimation (Vogel et al., 2014) to developmental responses such as flower pedicel abscission (Nakano et al., 2014), leaf senescence (Koyama et al., 2013), cell proliferation and shoot branching (Mehrnia et al., 2013). Other responses regulated by this superfamily include responses to biotic and abiotic stress, and overlapping responses to multiple stress factors (Jung et al., 2007). Originally considered to be plant-specific (Riechmann et al., 2000; Gutterson and Reuber, 2004), the AP2/ERF superfamily is now thought to have evolved through lateral gene transfer from bacteria and viruses into plants (Licausi et al., 2013).

The AP2/ERF superfamily is characterized by the AP2 domain, a conserved 60–70 amino acid DNA binding domain (DBD), which was initially identified in homeotic genes regulating flower development in *Arabidopsis thaliana* (Jofuku et al., 1994). Based on the number of AP2 domains, exon-intron structure and additional conserved protein motifs in *Arabidopsis* and rice, the AP2/ERF superfamily is subdivided into the following three families: ERF, AP2, and RAV (Nakano et al., 2006). Other reviews (Mizoi et al., 2012; Licausi et al., 2013) summarize the basis of this classification in detail. Unless stated otherwise, ERFs in this review refer to members of the ERF family.

The ERF family is further subdivided into the ERF and the C-repeat-binding factor/dehydration-responsive-element–binding protein (CBF/DREB) subfamilies based on different conserved amino acid residues within their respective AP2 domains (Sakuma et al., 2002; Nakano et al., 2006; Lata and Prasad, 2011). The amino acid sequence variation in the AP2 domains of ERF and DREB subfamily members reflects the differences in the DNA binding specificities of these TFs and forms the basis for conditional responses regulating different sets of stress-responsive genes. In dicotyledonous plants, CBF/DREB proteins bind to the C-repeat/dehydration-responsive-element (CRT/DRE; A/GCCGAC) *cis*-acting element in the promoters of target genes and are usually associated with responses to abiotic stress and the phytohormone abscisic acid (ABA; Sakuma et al., 2002; Licausi et al., 2013). ERF subfamily members bind to the GCC-box (AGCCGCC) and are associated with biotic stress responses, responses to the phytohormones jasmonic acid (JA) and ethylene (ET), wounding, and development (Sakuma et al., 2002). Several exceptions to this broad generalization exist. *Arabidopsis thaliana* (*At*) ERF1 (ERF-subfamily group III A-4) and *At*TINY (DREB subfamily group IX B3; Nakano et al., 2006) are each capable of binding to both GCC and CRT/DRE elements, binding to the GCC box at the promoters of biotic stress-inducible genes and to the CRT/DRE elements of genes associated with abiotic stress tolerance thereby possibly balancing responses to biotic and abiotic stress conditions (Sun et al., 2008; Cheng et al., 2013). Also, ERF TFs of the subgroup IXA of the ERF subfamily in *Arabidopsis*, *Nicotiana tabacum*, and *Catharanthus roseus* contain fully conserved AP2 domains, but recognize distinct DNA sequences (Shoji et al., 2013). Taken together, although promoter motifs defining ERF and CBF/DREB-specific binding have been identified, both divergence and crosstalk of binding capacities exist, possibly promoting plasticity in stress tolerance.

In monocotyledonous plants, studies on the ERF family conducted over the last decade have mostly concentrated on the heterologous expression of *Arabidopsis* family members in crop plants and *vice versa*. Interestingly, most of these reports reveal augmented stress tolerance under transgenic conditions in comparison with wild-type plants. These findings suggest that the mechanisms for the translation of ERF function and their downstream targets are conserved between dicots and monocots. Here, we summarize current knowledge on the regulation and biological roles of members of the ERF family of AP2/ERF TFs in monocotyledonous plants.

## The Monocot ERF Regulatome and Multistep Control of Stress Responses

The number of AP2/ERF TF genes that has been identified in monocot genomes ranges from 53 in barley (*Hordeum vulgare*; Hv) to 184 in maize (*Zea mays*; Zm; Table 1; Zhuang et al., 2011a; Du et al., 2014). Similar to AP2/ERFs in *Arabidopsis*, immense diversification of the AP2/ERF superfamily in monocots is observed with a clear dominance of the ERF family (Table 1). Bioinformatic studies on the chromosomal clustering of genes in monocots showed that segmental and tandem duplication events have led to the evolution and extensive diversification of the AP2/ERF superfamily (Sharoni et al., 2011; Lata et al., 2014), while polyploidy adds to increased complexity in wheat (Zhuang et al., 2011b). Moreover, extensive synteny exists within monocots, which is restricted by comparison in *Arabidopsis* (Rashid et al., 2012).

**TABLE 1:** **Summary of ERF family members in monocots**.

**Plant**	**Total number of AP2/ERF TFs identified**	**ERF family**	**Percentage ERF family**	**DREB subfamily members of the ERF family**	**ERF subfamily members of the ERF family**	**References**
Rice	164	113	68.9	52	79	Nakano et al. (2006)
Wheat	117	104	88.8	57	47	Zhuang et al. (2011b)
Maize	184	158	85.8	51	107	Du et al. (2014)
Foxtail millet	171	138	80.7	48	90	Lata et al. (2014)
Sorghum	126	105	83.3	52	53	Yan et al. (2013)
Barley	53	40	75.4	18	22	Zhuang et al. (2011a)

**FIGURE 1:**
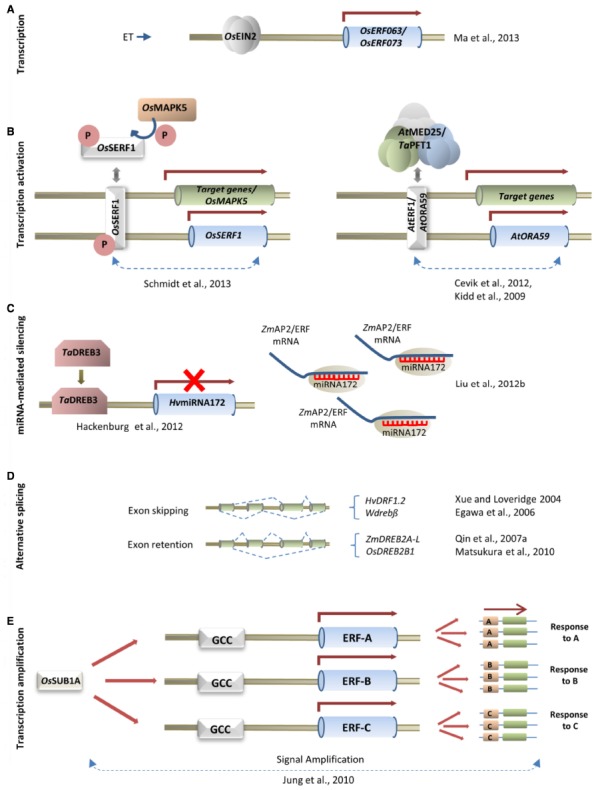
**ERF regulation at multiple levels to confer stress tolerance in monocots. (A)** Transcription: *OsERF063* and *OsERF073* transcript accumulation in rice is induced by ethylene (ET) in an *OsEIN2*-dependent manner. **(B)** Transcription activation. On left: Stress-induced phosphorylation of *Os*SERF1 by *Os*MAPK5 in rice produces the active ERF conformation facilitating DNA binding and thereby transcriptional activation of downstream genes, including *OsMAPK5*, and the ERF *Os*SERF1 itself in a positive feedback loop as indicated by the dotted line. On right: ERF transcription might be activated by the concerted action of the mediator complex (*At*MED25/*Ta*PFT1) binding to both AtERF1 and *At*ORA59 triggering target gene expression as well as *AtORA59* transcript accumulation in a positive feedback loop that is indicated by the dotted line. **(C)** miRNA-mediated silencing: barley miRNA172 is repressed by *TaDREB3* while miRNA172 in maize targets at least two AP2/ERF transcripts; the data suggest that miRNA172 acts both downstream and upstream of AP2/ERFs to regulate AP2/ERF transcript accumulation. **(D)** Alternative splicing: mRNA abundance of different *DREB2* isoforms in barley, wheat, maize and rice is controlled by stress-induced alternative splicing. **(E)** Transcription amplification: Signal amplification by (*Os*SUB1A) that induces downstream *ERF*s (Jung et al., 2010) possibly by binding to upstream GCC box in the promoters of the other ERF genes. This in turn leads to the expression of specific stress responsive downstream targets (A–C). Abbreviations: miRNA, microRNA; *TaDREB3*, *Triticum aestivum Dehydration Responsive Element Binding 3*; AP2/ERF, APETALA2/ETHYLENE RESPONSIVE FACTOR; *OsERF*, *Oryza sativa Ethylene Responsive Factor*; *OsEIN2*, *Oryza sativa Ethylene Insensitive 2*; *At*MED25, *Arabidopsis thaliana* MEDIATOR 25; *Ta*PFT1, *Triticum aestivum* PHYTOCHROME AND FLOWERING TIME 1; *AtERF1*, *Arabidopsis thaliana Ethylene Responsive Factor 1*; *AtORA59*, *Arabidopsis thaliana OCTADECANOID-RESPONSIVE ARABIDOPSIS APETALA2/ETHYLENE RESPONSE FACTOR domain protein59*; *Os*SERF1, *Oryza sativa* SALT-RESPONSIVE-ERF1; *Os*MAPK5, *Oryza sativa* MITOGEN-ACTIVATED PROTEIN KINASE5, *Os*SUB1A, *Oryza sativa* SUBMERGENCE1 locus gene A; GCC box, (AGCCGCC) *cis*-acting element; TFs, Transcription factors.

Despite being etymologically characterized as ET-responsive, ERFs can also be regulated in a manner independent of ET, by stress or by phytohormones including JA and ABA. In addition, transcriptional activators of the ET signaling pathway, including EHTYLENE INSENSITIVE2 (EIN2) may induce the expression of ERFs. For example, ET induces the expression of the rice (*Oryza sativa*; Os) *OsERF063* and *OsERF073* in an *OsEIN2*-dependent manner (Figure 1A; Ma et al., 2013). In general, the molecular regulation of gene expression relies on various steps of transcriptional activation/repression and post transcriptional and post translational control. While some information of transcriptional/post transcriptional control of ERFs in monocots is available (Figure 1), the involvement of post-translation control of ERFs in monocots remains mostly to be elucidated. The only clear evidence concerns the activation of rice SALT-RESPONSIVE-ERF1 (*Os*SERF1) upon its phosphorylation by MITOGEN-ACTIVATED PROTEIN KINASE5 (*Os*MAPK5). *Os*SERF1 phosphorylation enhances the transcription of downstream targets that include *OsMAPK5* and *OsSERF1* in a positive feedback loop (Schmidt et al., 2013; Figure 1B).

A recently identified component of the transcriptional machinery, the mediator complex is a large multi-protein complex that forms a central transcriptional co-regulator (Bäckström et al., 2007). It binds to gene promoter motifs forming a bridge to facilitate binding of TFs and RNA polymerase II. The mediator complex consists of about 20–30 subunits and is conserved in eukaryotes from yeast to plants and humans. The *Arabidopsis* MEDIATOR25 (*At*MED25) functions as a positive regulator of JA-dependent gene expression (Kidd et al., 2009; Cevik et al., 2012). It interacts with four group-IX ERFs, including *AtERF1* and *Arabidopsis OCTADECANOID-RESPONSIVE ARABIDOPSIS APETALA2/ETHYLENE RESPONSE FACTOR domain protein59* (*AtORA59*), at the promoters of these genes. *At*ERF1 and *At*ORA59, in turn, bind to the *At*ORA59 promoter (Zander et al., 2014) resulting in amplified expression of the downstream targets of *At*ORA59 (Cevik et al., 2012; Zander et al., 2014). The wheat (*Triticum aestivum*; Ta) *MED25* homolog *PHYTOCHROME AND FLOWERING TIME 1* (*TaPFT1*), when expressed constitutively in *Arabidopsis*, fully complements the *Arabidopsis Atmed25* phenotype. This result suggests a conserved function for MED25 in *Arabidopsis* and wheat (Kidd et al., 2009; Figure 1B).

Other studies suggest a role for miRNAs (20–24 nucleotide non-coding RNAs that post-transcriptionally repress gene expression) in the regulation of AP2/ERFs. Maize *Zm*-miRNA28 is associated with germination and predicted to target the ERF-like gene TC313437 (Ding et al., 2013). Another example in maize is related to abiotic stress. Waterlogging due to floods or sudden rains causes hypoxia of roots leading to anaerobic stress-induced metabolic changes and the accumulation of reactive oxygen species. Studies on short term waterlogging in two maize lines revealed the up regulation of *Zm*-microRNA172 (*Zm*-miRNA172), which targets the repression of the AP2/ERF TFs ZM5G862109 and ZM2G076602 (Liu et al., 2012b). Interestingly, miRNA172 was also identified in barley among miRNAs that were down-regulated by the heterologous expression of wheat *DREB3* (*TaDREB3*; Hackenberg et al., 2012). *TaDREB3* overexpression in barley resulted in robust drought-tolerant plants in comparison to severely wilted wild type plants on drought stress. The *Ta*DREB3-dependent downregulation of *Hv*-miRNA172 has been associated with the regulation of the expression of AP2/ERFs of the AP2 subfamily (Hackenberg et al., 2012). Taken together, miRNA172 represents a versatile regulatory node both targeting the expression of AP2/ERFs or itself being targeted by AP2/ERF TFs such as *Ta*DREB3 (Figure 1C).

Alternative splicing has been reported to play a major role in the stress-induced transcriptome of plants, with housekeeping genes undergoing normal splicing and genes encoding stress-related proteins over-represented in the alternatively spliced fraction (Li et al., 2013). Interestingly, the *DREB2*-like barley gene *DEHYDRATION RESPONSIVE FACTOR1* (*HvDRF1*) and its orthologs in wheat (*TaDRF1/Wdreb2*), maize (*ZmDREB2A*) and rice (*OsDREB2B*) undergo alternative splicing by differential exon usage (Xue and Loveridge, 2004; Egawa et al., 2006; Qin et al., 2007a; Matsukura et al., 2010). While the inactive forms of *HvDRF1* (*HvDRF1.2*) and *Wdreb2* (*Wdrebß*) originate due to exon skipping causing a premature termination before the AP2 sequence (Xue and Loveridge, 2004; Egawa et al., 2006), the inactive forms of *OsDREB2B* (*OsDREB2B1*) and *ZmDREB2A* (*ZmDREB2A-L*) are similarly terminated prematurely due to frameshift caused by a 53 bp exon retention (Qin et al., 2007a; Matsukura et al., 2010; Figure 1D). *HvDRF1* produces two active ERF isoforms (*HvDRF1.1* and *HvDRF1.3*) associated with ABA dependent drought tolerance. The wheat gene *Wdreb2* produces three isoforms, *Wdrebα*, *Wdrebß*, and *Wdrebγ*, which are homologous to *HvDRF1.1*, *HvDRF1.2*, and *HvDRF1.3*, respectively. The accumulation of *Wdrebα* and *Wdrebγ* transcripts is transient and more pronounced during drought/salt stress, while all three forms are induced by low temperature (Egawa et al., 2006). Likewise, the active isoform of the maize gene *ZmDREB2A*, *ZmDREB2A-S*, is significantly induced by drought and heat stress (Qin et al., 2007a). In rice, only the functionally active splice isoform of *OsDREB2B* (*OsDREB2B2*) is induced by low temperature stress (Matsukura et al., 2010). Taken together, alternative splicing allows isoform-specific transcript level adjustments regulating responses to normal and stress-induced conditions (Xue and Loveridge, 2004).

## ERFs in Monocotyledonous Plants: Convergent Biotic and Abiotic Stress-responsive Nodes

ERFs are versatile TFs that are involved in development as well as responses to different types of stresses. Often, ERF TFs affect cross talk between different responses acting at the interface between different signaling cascades. In *Arabidopsis*, for example, *At*ERF1 binds to different promoter elements to regulate responses to pathogen resistance (by binding to the GCC box) and to drought, salt and heat stress (by binding to the DRE/CRT element; Cheng et al., 2013). Similarly, *Ta*ERF1 binds to both the GCC box and the DRE/CRT elements, regulating responses to both biotic and abiotic stress in wheat (Xu et al., 2007). Several ERF subfamily members in monocots are associated with development and responses to biotic and abiotic stress, whereas CBF/DREB subfamily members to our present knowledge appear mostly related to responses to abiotic stress.

### The ERF Subfamily: Stress and Developmental Responses

Several monocot ERFs of the ERF subfamily are induced in response to development and biotic stresses, occasionally imparting multiple stress tolerance upon heterologous expression. The ectopic expression of the barley ERF gene *Hv ROOT ABUNDANT FACTOR* (*HvRAF*) in *Arabidopsis* promotes resistance to *Ralstonia solanacearum* and enhances tolerance to salt stress (Jung et al., 2007). In addition, constitutive expression of the wheat ERF genes, *TaERF1* in *Arabidopsis* and *TaERF3* in wheat enhances tolerance to salt and drought stress (Xu et al., 2007; Rong et al., 2014), while the *PATHOGEN-INDUCED ERF1* (*TaPIE1*) increases freezing stress tolerance in wheat (Zhu et al., 2014). Conversely, *TaERF4* acts as a negative regulator of salinity tolerance when constitutively expressed in *Arabidopsis* (Dong et al., 2012). Furthermore, *TaERF1* overexpression enhances tolerance to the necrotrophic pathogen *Botrytis cinerea* in *Arabidopsis* and to *Pseudomonas syringae* pathovar *tabaci* in tobacco compared to the respective wild type conditions (Xu et al., 2007). In response to the powdery mildew fungus *Blumeria graminis*, *TaERF3* is induced in a SA-dependent manner in a powdery mildew resistant wheat cultivar (Zhang et al., 2007). The same gene is induced by methyl jasmonate/ET, which is correlated with resistance against the necrotrophic pathogens *Fusarium graminearum* and *Rhizoctonia cerealis* (Zhang et al., 2007). The involvement of ERFs in wheat resistance against *R. cerealis* was further supported by evidence of enhanced resistance to this pathogen in transgenic *TaPIE1* over expressing wheat plants (Zhu et al., 2014).

Transcript abundance of ERFs may contribute to condition-specific responses. For example, the overexpression of the rice gene *OsSERF1* in rice leads to salt tolerance (Schmidt et al., 2013) and knockdown of this gene leads to developmental responses such as grain filling. *Os*SERF1 positively regulates the transcription of target genes, including the MAP kinase kinase *OsMAP3K6* and *OsMAPK5* (Figure 1B) and other TFs, such as *OsDREB2A* and negatively regulates *RICE PROLAMIN-BOX BINDING FACTOR* (RPBF) which is responsible for grain filling (Schmidt et al., 2014). Several other rice ERFs contribute to developmental or biotic stress responses either positively or negatively. While overexpression of the rice ERF *OsERF1* in *Arabidopsis* resulted in stunted growth and increased accumulation of ET responsive genes (Hu et al., 2008), overexpression of another ERF *OsEATB* (for *Oryza sativa* ERF protein associated with tillering and panicle branching) resulted in reduced plant height but higher tillering, thereby increasing yield potential (Qi et al., 2011). Also, overexpression of the *SNORKEL1* (*OsSK1*) and *SNORKEL2* (*OsSK2*) genes, which encode ERFs homologous to *AtERF1*, induces elongated internodes in an ET-dependent manner in rice (Hattori et al., 2009). Finally, *OsERF922* is a negative regulator of defense against the rice blast fungus *Magnaporthe oryzae* and salt stress (Liu et al., 2012a). Interestingly, *OsERF922* overexpression enhanced ABA levels in rice, which might be causative for the enhanced susceptibility to *M. oryzae*, as exogenous ABA treatment also induces susceptibility to this pathogen (Koga et al., 2004).

A few ERFs have been associated with long-distance leaf-to-leaf and root-to-leaf signaling in monocots to induce a state of potentiation/priming against future pathogen attack. Treatment of rice roots with the *Pseudomonas* isolate EA105 induces the expression of *OsERF1* in distal uninfected leaves and enhances resistance to *M. oryzae* compared to non-infected plants (Spence et al., 2014). Similarly, we related two previously uncharacterized ERFs, *HvERF-like* and *HvERF_44411*, to leaf-to-leaf long-distance signaling in barley systemic immunity. Transcript accumulation of both of the ERFs identified was induced in barley leaves that were infected with *Pseudomonas syringae* pathovar *japonica* or *Xanthomonas translucens* pathovar *cerealis* and in systemic, uninfected leaves of the infected plants (Dey et al., 2014), possibly priming the systemic leaves for rapid transcriptional reprogramming upon further pathogen challenge.

### The CBF/DREB Subfamily: Responses to Cold, Drought and Salinity

The abiotic stress-responsive CBF/DREBs highlight the plasticity of the ERF family in multiple stress tolerance. Divided into two subgroups, CBF/DREB1 and DREB2, the CBF/DREB1 members are broadly associated with freezing tolerance and the DREB2s with dehydration and salt stress responses (Choi et al., 2002; Mizoi et al., 2012). Cross talk between these responses has been observed (Oh et al., 2007; Matsukura et al., 2010), which may be partly attributed to a common subset of stress-inducible downstream targets in addition to TF-specific target genes depending on the initial stress response. Target genes of non-cold-inducible *CBF/DREB1s*, for example, impart drought tolerance and overlap with those that are induced by cold-inducible *CBF/DREB1s* (Mizoi et al., 2012). The barley cold-responsive genes *HvCBF1* (*Hordeum vulgare* C-repeat-binding-factor 1), *HvCBF2*, and *HvCBF3* are transcriptionally induced by chilling (Choi et al., 2002; Xue, 2003). Low temperature induces the active conformation of *HvCBF2*, thereby facilitating DNA binding. HvCBF2 thus represents a TF that undergoes temperature-sensitive changes that result in an increased TF-DNA binding affinity and the transcriptional activation of target genes (Xue, 2003). The rice genes *OsDREB1A* and *OsDREB1B* are induced by cold and *OsDREB2A* by dehydration and salinity. However, overexpression of *OsDREB1A* alone enhanced tolerance to drought, salinity and freezing in both *Arabidopsis* and rice (Dubouzet et al., 2003; Ito et al., 2006), while *OsDREB1B1* increased tolerance to both high and low-temperature stress in transgenic *Arabidopsis* (Qin et al., 2007b). Individual ectopic expression of the wheat *CBF/DREB1s TaCBF14* and *TaCBF15*, in barley improved tolerance to freezing (Soltész et al., 2013).

## ERFs: A Biotechnological Toolkit for Signal Amplification and Multiple Stress Tolerance

In several cases, the robust stress-tolerant phenotypes of plants that over express heterologous ERFs may result in part from signal amplification via the induction of further endogenous ERFs added to other stress-inducible genes. For example, the constitutive expression of *TaDREB2* and of *TaDREB3* in barley induced tolerance to freezing and drought stress and the endogenous expression of the barley CBF/DREB factors *HvCBF1*, *HvCBF3*, *HvCBF6*, *HvCBF10A*, *HvCBF11*, *HvCBF15*, and *HvCBF16*, in addition to other stress-responsive genes (Morran et al., 2011). The CRT/DRE elements in the promoter regions of CBF/DREB genes could provide an explanation for the robust amplification of stress signals (Soltész et al., 2013). The role of ERFs in the amplification/multiplicity of stress signals is also notable in rice (Jung et al., 2010; Wan et al., 2011; Zhang et al., 2013). *DROUGHT-INDUCED ERF1* (*OsDERF1*) is induced by drought, ET and ABA, while rice plants overexpressing *OsDERF1* show enhanced sensitivity to drought. An opposite phenotype is observed in knockdown plants, revealing that OsDERF1 is a negative regulator of drought tolerance in rice (Wan et al., 2011). Notably, the genes that are activated by OsDERF1 include two ERFs, *OsERF3* and *OsAP2-39* (Wan et al., 2011). The hierarchal concept of an upstream ERF regulating multiple other ERFs is also observed for *SUBMERGENCE1A* (*OsSUB1A*) that is one of three ERFs constituting the Submergence1 (*Sub1*) locus in rice. *Os*SUB1A plays a pivotal role in the recovery of submerged rice plants and is associated with negative regulation of gibberellic acid responsiveness and ET production. Overexpression of *OsSUB1A* results in increased ABA responsiveness and tolerance to oxidative stress (Fukao et al., 2011). Furthermore, comparative transcriptome analysis of submergence-intolerant and submergence-tolerant, *OsSUB1A*-containing rice lines revealed an *OsSUB1A*-dependent regulation of 12 target ERFs. Further analysis allowed the classification of these ERFs into three distinct functional categories, thereby controlling different biological aspects of stress tolerance (Jung et al., 2010; Figure 1E).

## Conclusion and Perspectives

The TFs of the ERF family are among the most versatile stress-responsive TFs in plants. The extensive plasticity of ERFs in inducing multiple stress tolerance in heterologous plants is perhaps the greatest motivation for exploiting the family for plant biotechnology in light of the changing global climate. While ERFs from newly sequenced crop plants continue to be identified, it will be interesting to further characterize their role at the functional level. Moreover, large-scale transcriptome co-expression analyses may provide a basis for identifying upstream regulatory ‘hub’ ERFs. “Hub” ERFs might activate downstream TFs, including other ERFs, which may in turn lead to signal amplification and multiple stress tolerance. It will further be essential to correlate ERFs with their target *cis*-elements in different crop plants and to characterize specific and potentially non-specific gene expression changes upon ERF over expression and their effects on the robustness of the plant under stress. With this knowledge, new strategies may be designed to engineer multiple stress tolerance in crops.

### Conflict of Interest Statement

The authors declare that the research was conducted in the absence of any commercial or financial relationships that could be construed as a potential conflict of interest.
